# Risk assessment of methanol storage tank fire accident using hybrid FTA-SPA

**DOI:** 10.1371/journal.pone.0282657

**Published:** 2023-03-08

**Authors:** Ehsan Ramezanifar, Kamran Gholamizadeh, Iraj Mohammadfam, Mostafa Mirzaei Aliabadi

**Affiliations:** Center of Excellence for Occupational Health, Occupational Health and Safety Research Center, School of Public Health, Hamadan University of Medical Sciences, Hamadan, Iran; University of Catania, ITALY

## Abstract

Fire accidents in storage tanks are of great importance due to the difficulty in extinguishing and ease of spread to nearby products. This study aimed to introduce a framework based on FTA-based Set Pair Analysis (SPA) established via experts’ elicitation to identify and assess the risk of storage tank fire. In the quantitative FTA of a system, sufficient data are only sometimes available to calculate the failure probability of the system appertains to study. Thus, the obtained result of the SPA added new value to the Basic Events (BEs) and estimated top event. To illustrate the applicability of the proposed approach, a fault tree of the methanol storage tank fire is performed and analyzed BEs. According to the obtained results, the fire accident was computed by 48 BEs, and the occurrence probability value of the top event was estimated 2.58E-1/year. In addition, the most crucial paths that led to the fire accident are listed in this study. The proposed approach established in the present study can assist decision-makers in determining where to take preventative or appropriate action on the storage tank system. Moreover, it can be adjusted for various systems with limited manipulation.

## Introduction

Nowadays, with the growth in demand and production of methanol, a versatile fuel with the potential to become an alternative fuel, more available storage is required [1]. This combustible commodity has an increasing production trend, and poses a global consumption of approximately 92Mt/year [2, 3].

Due to the steady growth in chemical storage, the problems of fire accidents in storage tanks continue to rise [4, 5]. According to the literature, Fire accidents were the most recurring in significant installations such as petroleum refineries, oil terminals, or storage parks which threaten life, the environment and public opinion [6–8]. Although most companies are advised to follow engineering guidelines or standards for safety measures allocation in order to storage fire risk reduction, they cannot prevent fire, and the possibility exists for various causes [9–11]. For example, in June 2021, a storage fire accident occurred in the Tehran oil refinery due to leakage. The fire spread to nearby products and equipment, which caused to series of secondary fires, considerable property damage and a serious concern for local people [5, 12].

Many storage fire accidents have occurred throughout history, as evidenced by the Bunce field fire and the Jaipur oil depot fire that have caused severe environmental pollution, substantial economic losses and direct tens of casualties [13, 14]. These catastrophic incidents mainly rely on two possible causes: the inherent nature of chemicals and the involved quantity stored on-site [15, 16]. In that sense, the safe operation of storage tanks while mitigating the fire consequences has become more challenging. On the other side, preventing and having a plan for dealing with such catastrophic incidents is a logical solution to reduce casualties and property losses while contributing to social stability and environmental concerns with respect to something humans value [17, 18]. Therefore, conducting a thorough and effective risk assessment of the storage tank is crucial to describe and identify risks more accurately.

Risk assessment has been accepted as a well-known way to predict and reduce the likelihood of adverse events regarding safety on industrial sites [19, 20]. Among risk assessment techniques, Fault Tree Analysis (FTA) is excellent at presenting how the system resists singular or numerous initiating faults [21, 22]. Since the creation of FTA, it has been explored in many fields, including marine [23], oil and gas [24], petrochemical processes [25], refineries [26], nuclear power plants [27], and Hazmat transportation [16, 28] which show that it can be a suited technique to reveal the fundamental contributing factors to an undesirable event correctly [29]. FTA has multiple applications in assessing safety, security and reliability at different phases of the system, but it has some drawbacks [9]. For instance, determining quantitative failure probability is one of the main challenges. Using this technique on a quantitative scale requires full access to the failure data. It is also limited to some events caused by subjective and uncertain factors such as human behaviour [30, 31].

Because of the difficulties stated above, results from FTA-based risk assessment could be questioned and may need to be improved in the accuracy of the results and system analysis. Some studies addressed this issue in various kinds of complexity approaches, including the Bayesian network [32–35], Monte Carlo [36], the Petri net [37] and the Fuzzy set theory [24, 30, 38], etc. Yazdi et al. [39] used the intuitionistic fuzzy technique in the consensus of experts to tackle the uncertainty in estimating the FTA probabilities. Existing research discloses the most common approaches to reducing uncertainty through FTA limitation management, but they have intricated and time-consuming. To that end, another method that has recently become increasingly familiar to scholars, user-friendly and less complex is Set Pair Analysis (SPA) [40–43].

SPA is a mathematical theory to describing uncertain problems with a clear concept and implementation. It is a numerical method based on hybrid certainty-uncertainty into a single framework and incorporates expert judgments [40–42]. Over the last decade, researchers have used some assessments [41, 44, 45] and decision-making [46] to demonstrate the SPA’s ability to deal with uncertain problems [47]. Based on the combination of SPA and FTA, Zhao et al. [48] studied the current leak fault diagnostic for the launch vehicle. In addition, Xue-hui and Li-xing [49] employed FTA for the safety evaluation of the tailing pond and then introduced the SPA theory into the FTA to determine the occurrence probability of top events. With others focusing on its features, SPA was selected for its simple calculation that can be easily applied to the fault tree.

To the best of our knowledge, the integrated methodology of FTA and SPA has not been employed to assess storage tank fires. Given this, the current study adopts the proper assessment method to determine the failure probability of the storage fire accident. The secondary objective of this study focused on ‘how to obtain failure probability of the system once its components have no quantitative failure data’. The proposed approach could be effectively applied to evaluate and manage storage fire risks in diverse industries.

To sum up, the paper’s organization is followed. Section 2 reviews the proposed approach. This section includes some basic definitions useful for the present study. In order to indicate the feasibility and effectiveness of the proposed approach, a quantitative storage fire risk assessment and its main steps are executed in Section 3. Finally, in Section 4 are summarized feature remarks and conclusions.

## Methodology

**Fig 1:**
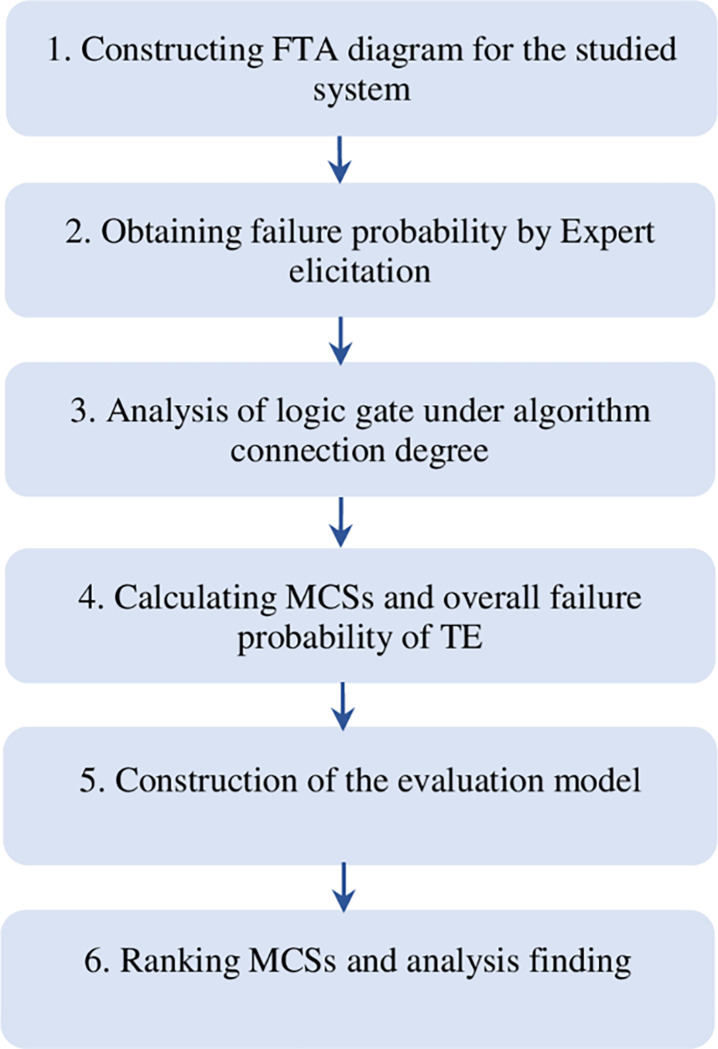
Evaluation flowchart based on the model of FTA-SPA.

The proposed methodology and its flowchart are represented in detail, as illustrated in Fig 1.

### Ethical statement

The study was approved by the ethics committee of Hamadan University of Medical Sciences (Ethics Committee No: IR.UMSHA.REC.1400.189).

### Root cause analysis

There are usually several causes behind any problem [50]. Identifying and eliminating those causes are perceived to be in a position to prevent the problem from persisting [51]. As a fault diagnosis procedure, root cause analysis aims to identify the origin cause of faults [52, 53].

One of the analytical tools is deductive FTA which is broadly employed to clarify the direct and indirect root causes [54].

### Fault Tree Analysis (FTA)

FTA is a properly systematized technique for assessing the failure probability of different systems into a visual diagram called a fault tree, which allows both quantitative and qualitative evaluation projects [22, 55]. An FTA is the backward safety analysis method that begins with the possible dangerous event (top event or TE) and progresses to the Intermediate Events (IEs) and Basic Events (BEs), respectively [56]. The FTA can be constructed by two nodes (gate/event) and the graphical decomposition of a TE into IEs and BEs articulated by logic gates [9].

IEs are explained in greater detail until BEs or undeveloped events are discovered. BE is an initiating or basic fault that requires no further elaboration because a sufficient degree of resolution has been attained. Each IE is a fault due to the logical combinations of different events occurring further down the tree [55]. Countless inputs can be used in logic gates. Intermediate, basic, undeveloped events and gates (AND/OR) can all be used as inputs. If one or several inputs occur, the OR gate’s outcome happens. Generally, an AND gate’s output event happens only if all input events occur together [23]. BEs combine through the logic gate to cause and end IEs and top events.

#### Determination of failure probability of basic events

The next step is to compute the failure probability of BEs, which is usually required after creating the fault tree. BEs are often given available failure rates or repair rates to facilitate quantitative analysis. When failure rates are already established and available, failure probability would be obtained easily from Eq (1) and Eq (2). It is worth noting that failure rates are inspectable can be performed as Eq (3) [25].


$$FP(t)=1-e^{-\lambda t},$$ (1)

FP(t)=λt,(λt<0.1) (2)

FP(t)=(λτ)/2,(λτ>0.1) (3)

Here, *FP*(*t*) is the failure probability of the event, *λ* expresses the failure rate, *t* is the time used for the experiment and *τ* indicated as the interval of inspection.

However, as long as the failure rates to measure the failure probability of BEs are un-clear and absence of data, there are difficulties in determining the failure probability of TE, which leaves us with ambiguity. For example, no failure rates are available for the drain valve supplied on the fault tree of a storage tank. In such a situation, experts could be consulted to meet these difficulties. This research addresses this issue. To overcome these limitations and quantify the failure probability of the BEs, as previously indicated, one approach that elicits expert judgment and has recently found a wide range of applications is SPA.

### Set Pair Analysis (SPA)

The SPA was initially developed in 1989 by Zhao KQ et al. [40]. It investigates certainty and uncertainty using three elements of the Identity, Discrepancy and Contradistinction model (IDC-model), and represents the relationship between two sets in its entirety. The main idea of SPA is to use a mathematical method directed toward determining uncertainty problems with intuitive evaluation. SPA inclusive dual interrelation sets have a particular Connection Degree (CD) [44, 57]. With this in mind, uncertainty is defined as a difference between two aspects of certainty: "same" and "opposite". Under controlled and specified conditions, they are interconnected, affected mutually, restrained and transformed by each other [47, 58]. Following this sub-section, the preliminary SPA theory is presented, and some of its features and concepts are discussed.

#### Preliminary

This stage covers some basic definitions that are appropriate for this study.

*Definition 1*. Let’s assume that the sets given were "*α*" and "*β*", a set pair is composed of two collections with links, i.e.: *μ* = (*α*,*β*). Both "*α*" and "*β*" have n items representing their characteristics, so = (*α*_1_, *α*_2_, *α*_3_,…,*α*_*n*_), *β* = (*β*_1_, *β*_2_, *β*_3_,…,*β*_*n*_). The concept of the CD expressing the relationship of *μ* = (*α*,*β*) is [59]:

$$\mu_{\alpha ∼\beta }=(S/n)+(F/n)i+(N/n)j=A+Bi+Cj$$ (4)

Here, *A* = (*S*/*n*), *B* = (*F*/*n*) and *C* = (*N*/*n*) denote the set pair’s identities, discrepancy and contradistinction degree. Moreover, *S*+*F*+*N* = *n*, i: represents the uncertainty coefficient and depending on different circumstances: *i*∈[−1, 1], j: represents the coefficient of opposition, and constant *j* = (−1). Noticing that *B* = 1−(*A*+*C*) and 0≤*A*,*B*,*C*≤1 [59].

In Eq (4), A reflects the degree to which sets "*α*" and "*β*" have the same property in terms of a certain attribute. B reflects the degree to which sets "α" and "β" have properties that are neither the same nor opposite of a certain property. C reflects the degree to which sets "*α*" and "*β*" are related the degree to which a certain attribute has the opposite nature. When "*i*" and "*j*" take reasonable values, {*μ*_*α*~*β*_} becomes a numerical value, which is denoted as {*μ*′_*α*~*β*_}. According to the definition of CD: −1≤*μ*′_*α*~*β*_≤+1.

*Definition 2*. Connection number and failure probability

By assuming the failure probability of each event *i* can be represented as *P*_*i*_ = (*A*_*i*_, *B*_*i*_, *C*_*i*_), the connection number related to the identified events through expert judgment should be assigned. Therefore, the failure probability of the BEs differs from the preceding ones. In general, three aspects (i.e., probability of non-occurrence (an event never happens), the probability that may or may not occur (medium state) and the probability of occurrence (without doubt, events happen)) are used to determine the subject’s failure probability [49, 60].

Since experts have different knowledge and views about the same subject (or BE), their judgment is weighted differently based on years of related experience, age, job title and educational level [61, 62]. In order to make evaluation results more in line, the criteria of weighting factors of experts can be determined as shown in Table 1 and Eq (5) [21, 63].

**Table 1:** Weighting scores of various experts [65].

Group	Classification	Score
Job title	Operator	1
	Technical	2
	Engineer	3
	Manager, Factory inspection	4
	Chief Engineer, Director	5
Educational level	High school	1
	Higher national diploma	2
	Bachelor	3
	Master	4
	PhD	5
Related experience (year)	≤ 5	1
	6 to 9	2
	10 to 19	3
	20 to 29	4
	≥ 30	5
Age	> 30	1
	30 to 39	2
	40 to 49	3
	≤50	4


$$W_{i}=\frac{The\;weigh\;score\;of\;i^{th}\;Expert}{\sum_{i=1}^{n}The\;weight\;score\;of\;Experts}$$ (5)

This weight is added to the matrix computation of the assessment subjects [64]. The following model (Eq (6)) can be used to describe it:

$$\mu (w)=W\cdot R\cdot E=(W_{1},W_{2},\ldots ,W_{n})\cdot [\begin{array}{ccc} A_{1} & B_{1} & C_{1}\\ A_{2} & B_{2} & C_{2}\\ \vdots & \vdots & \vdots \\ A_{n} & B_{n} & C_{n} \end{array}]\cdot [\begin{array}{l} 1\\ i\\ j \end{array}]=\sum_{s=1}^{n}W_{s}A_{s}+\sum_{s=1}^{n}W_{s}B_{s}i+\sum_{s=1}^{n}W_{s}C_{s}j$$ (6)

Here, *W* = (*W*_1_, *W*_2_,…,*W*_*n*_) refers to the weight vector-matrix of the different experts to assess subjects on each system, *R* refers to set on the vector-matrix of each subject’s evaluation, and *E* refers to set the coefficient matrix (or the connection degree matrix).

*Definition 3*. The evaluation model’s application

This definition aims to consider the risk level. To create an identities-discrepancy-contradistinction assessment level, suppose the connection degree related to *i* is [−1 to 1], and its range is split into three sub-intervals [−1,−0.333], [−0.333,0.333] and [0.333,1] according to the "equally" concept. The corresponding grades are "unacceptable condition", "medium state" and "acceptable condition" respectively [66].

*Definition 4*. Basic algorithm and application rule of CD

Sum algorithm rule. The connection number assigned to a subject result from the expert’s judgment. This algorithm is mainly used to aggregate two connection numbers. Therefore, by considering {*μ*_1_ = *A*_1_+*B*_1_*i*+*C*_1_*j*} and {*μ*_2_ = *A*_2_+*B*_2_*i*+*C*_2_*j*}, then the summation of two CDs follows these rules as Eq (7) [49]:

$$\mu_{1}+\mu_{2}=2\left[\frac{\left(A_{1}+A_{2}\right)}{2}+\frac{(B_{1}+B_{2})i}{2}+\frac{(C_{1}+C_{2})j}{2}\right]$$ (7)

Multiplication algorithm rule. According to the CD multiplication algorithm, we have {*i*×*j* = *i*, *j*^2^ = 1, *i*^2^ = *i*}, then the two CDs are multiplied as Eq (8):

$$\mu_{1}*\mu_{2}=(A_{1}A_{2}+C_{1}C_{2})+(1-(A_{1}A_{2}+C_{1}C_{2})-(A_{1}C_{2}+A_{2}C_{1}))i+(A_{1}C_{2}+A_{2}C_{1})j$$ (8)

### Combination of FTA and SPA

As mentioned earlier, the FTA uses logic gates symbols such as AND/OR to establish a logical relationship between BEs that ultimately contribute to TE. In order to compute the failure probability of the TE by considering the *BEs* = {*BE*_1_, *BE*_2_, *BE*_3_,…,*BE*_*n*_}, there are two procedures [49]:

The CD of the TE connected with the "AND gate" is estimated using Eq (9):

$$\mu_{\alpha ∼\beta }({BE}_{1}∙{BE}_{2}∙{BE}_{3}∙\ldots ∙{BE}_{n})=\prod_{m=1}^{n}\left(A_{m}+B_{m}i+C_{m}j\right)$$ (9)

The CD of the TE connected by "OR gate" is estimated using Eq (10):

$$\mu_{\alpha ∼\beta }({BE}_{1}+{BE}_{2}+{BE}_{3}+\ldots +{BE}_{n})=n\left(\frac{\sum_{m=1}^{n}A_{m}}{n}+\frac{\sum_{m=1}^{n}B_{m}}{n}i+\frac{\sum_{m=1}^{n}C_{m}}{n}j\right)$$ (10)

FTA provides the most crucial BEs with the path of system failure and subsequently, the SPA approach is used both to determine the failure probabilities of BEs and top event. Applying SPA as a supplement to FTA, particularly with inadequate data on BEs failure probability, makes safety evaluation important and becomes an enlargement of the systems guarantee.

### Minimum Cut Set (MSC) and TE calculation

Once the fault tree diagram is formed, quantitative and qualitative evaluations can begin. In qualitative evaluation, it is essential to determine the Minimum Cut Sets (MCSs) that are unique combinations of events or single events propagated upward to the top event. Another MCS application is to calculate the likelihood of the occurrence of ultimate events applied in quantitative evaluations. Each order related to MCSs consists of the number of BEs. For example, first-order poses single BE can lead to a top event. The second-order include two BE in each MCS, and the third-order has three BE can cause system failure. In general, the lower order related to MCS has more importance [55]. A fault tree often has a some MCS, from which the overall probability of the tree can be calculated.


$$TE={MCS}_{1}+{MCS}_{2}+\cdots +{MCS}_{n}$$ (11)

TE likelihood is as follow:

$$P(TE)=P({MCS}_{1}⋃{MCS}_{2}⋃\ldots ⋃{MCS}_{n})$$ (12)

### Ranking MCS in a fault tree

One reason for using FTA is that it can provide outputs to determine the importance measure set for calculating top events. Identifying each MCS’s importance can provide useful information for risk-related decision-making [65]. According to the calculated top event, the most crises MCS can be ranked by employing Fussell–Vesely Index (F-VI). In this study, F-VI performed to ranking MCS as follows:

$$I_{i}^{F-V}(x)={PC}_{i}(x)/P_{s}(x)$$ (13)

Where $I_{i}^{F-V}(x)$ is the importance of *MCS*_*i*_, *PC*_*i*_(*x*) represents the probability of *MCS*_*i*_ and *P*_*s*_(*x*) is the probability of failure of top event due to all MCSs.

Considering the above relationships and the connection number given to each BE, the FTA can be estimated differently. This approach tries to be the simplest form of direct prediction and enables us to obtain the failure probability. To demonstrate the applicability of the presented procedure in an industrial setting, a complete fault tree of the methanol storage tank fire is constructed and analyzed using the FTA-SPA.

## Application and results

**Fig 2:**
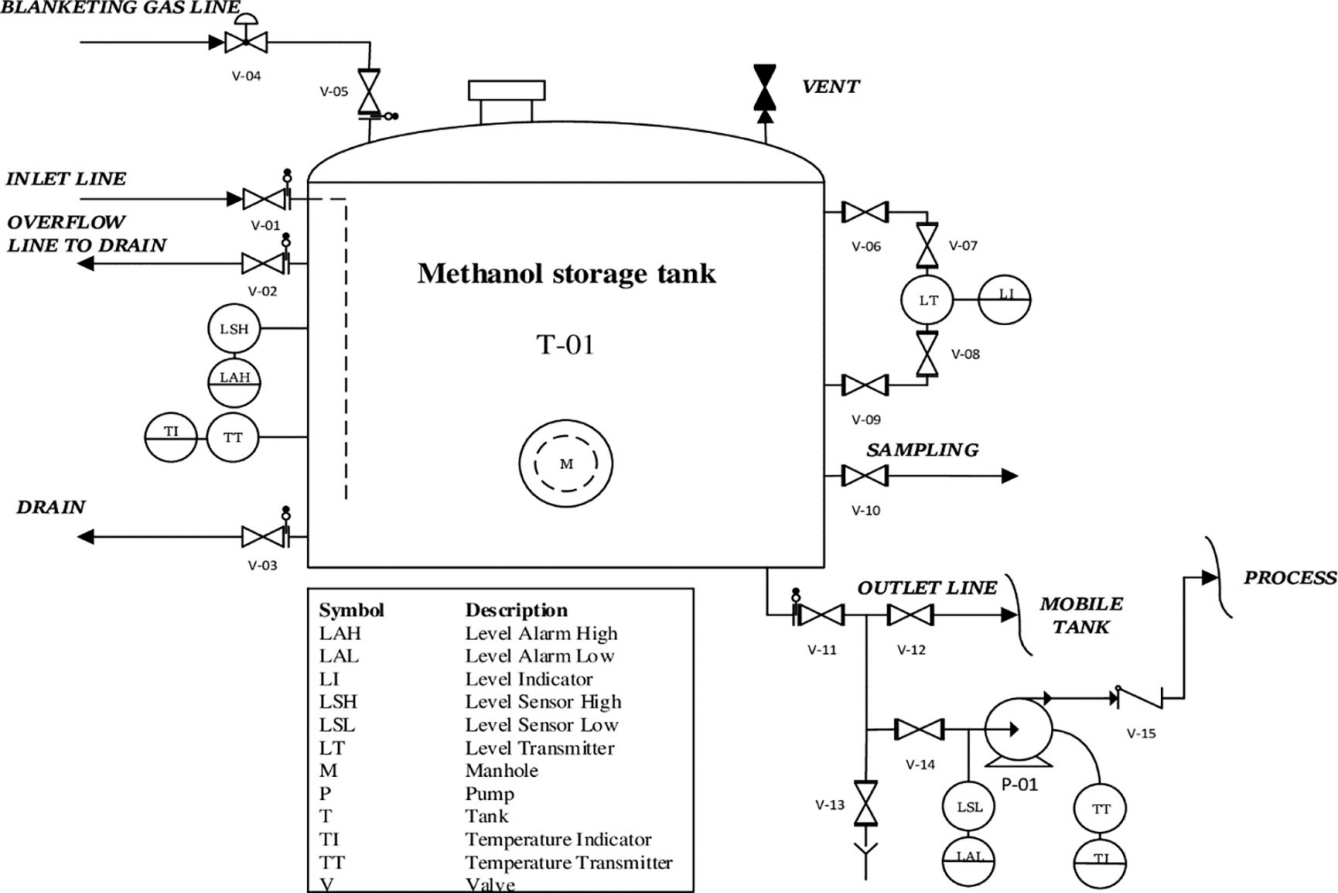
P&ID of cone roof tank with blanketing gas line.

Storage tank fire incidents are rare but can often induce massive consequences to the environment, asset and human safety. These features are one of the main reasons for investigating and have caused it to be noticed. Various types of storage tanks are found in most sectors of the petroleum industry to handle large quantities of liquids with high economic value [67]. Methanol storage tanks usually have fixed-cone roofs equipped with a nitrogen inerting system that operates at atmospheric pressure of less than 0.5 bar [68, 69]. Fig 2 shows a generic sample Piping and Instrumentation Diagram (P&ID) of the methanol tank design with the main inlet and outlet lines.

### Application and development of FTA

**Fig 3:**
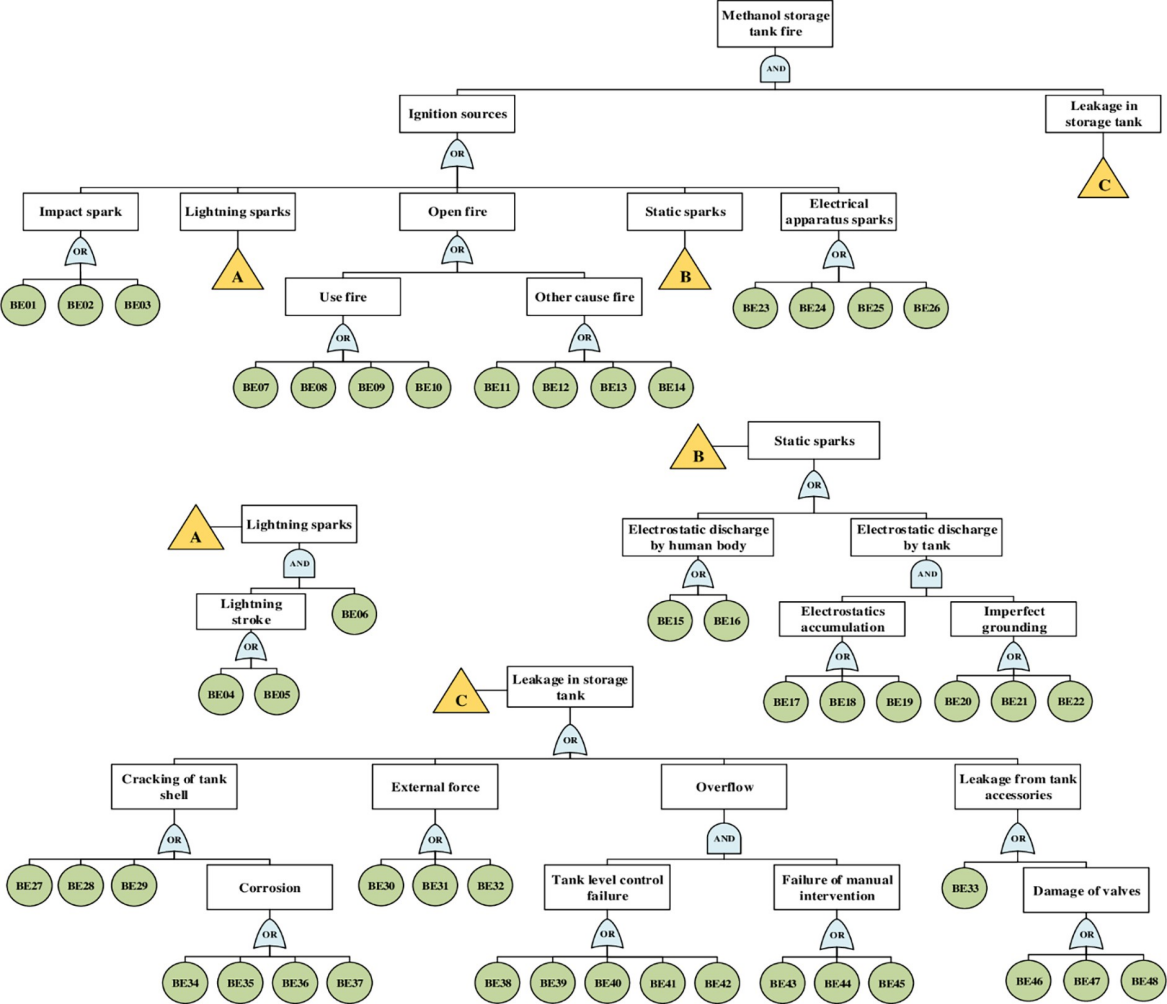
Fault tree of methanol storage tank fires accident.

Hazard identification should be recognized as an initiating point for evaluating systems. Before establishing the fault tree, hazard identification for potential triggers of storage tank fire was conducted based on operational experience, previous studies and maintenance data. To develop the proposed fault tree, a research team composed of seven well-versed experts familiar with the chosen system and currently active in this field was consulted. Accordingly, Fig 3 presents the BEs of the fault tree with the TE: fire accident in a methanol storage tank. The main description of BEs that may lead to this TE are listed in Table 2.

**Table 2:** Basic events in the proposed fault tree and descriptions.

Event	Description	Event	Description	Event	Description
BE01	Wearing iron nail-shoes	BE17	High flow velocity in loading and unloading	BE33	Poor seal around manhole
BE02	Collision of metal tools and tank wall	BE18	Measurement errors	BE34	Corrosion serious of the tank wall
BE03	Using non-explosion tools	BE19	The rough inner wall of the pipeline	BE35	Thermal stress
BE04	Direct lightning flash to storage tank	BE20	Non-standard apparatus	BE36	Lack of cathodic protection setting
BE05	Lightning flashes directly near pipelines and equipment	BE21	Non-standard ground resistance	BE37	Insufficient inspection and maintenance
BE06	Lack of lightning protection system	BE22	Ground wire damaged	BE38	Acoustic alarm failure (level alarm high / high-high)
BE07	Smoking	BE23	Mobile telephone	BE39	Level indicator failure (high / high-high)
BE08	Match	BE24	Other electronic devices	BE40	Level transmitter failure
BE09	Lighter	BE25	Sparks resulting from the welding process	BE41	Failure to close the automatic shut-off
BE10	Firework around the tank area	BE26	Non-explosion-proof equipment	BE42	Failure of the circuit breaker shutdown of the pump
BE11	Vehicles without flame arresters	BE27	Tank aging	BE43	The wrong valve opened
BE12	Using non-explosion lamps	BE28	Mechanical fatigue	BE44	Excessive loading
BE13	Dry grass	BE29	Poor welding quality	BE45	Failed to send the signal
BE14	Existence of heat sources near the farm tank	BE30	A natural disaster like an earthquake	BE46	Drain valve failure
BE15	The friction work	BE31	Catastrophic rupture by vehicle hit	BE47	UV-valve (on/off) failure
BE16	Wearing non-antistatic clothing	BE32	Terrorist attack	BE48	Sampling valve failure

Based on the previous paragraph, two IEs must coincide: "ignition sources" and "leakage in the storage tank". Therefore, they must be connected to the TE by an AND gate. As shown in Fig 3, possible ignition sources are impact sparks, static sparks, open fire, lighting sparks and electrical device sparks. Moreover, as any one of them could start a fire if encountered, they must be linked by an OR gate. According to the literature [69], lighting sparks, static electricity and open fires were the most critical cause of storage tank accidents. In the proposed fault tree, the causes of ignition sources include 26 BEs. Apart from ignition sources, determining the causes of leakage is essential, and the occurrence of leakage must be checked.

There are also various sources of leakage in the storage tank, including cracking tank shell, external force, overflow and leakage from tank accessories. Cracking tank shell involves corrosion causes, tank aging, mechanical fatigue and poor welding quality. Three causes of leakage (BE30, BE31 and BE32) due to external force are listed in Table 2. One of the main causes of leakage is the overflow that two IEs must occur together: "tank level control failure" and "failure of manual intervention". Among the causes of leakage, only this one should be connected by an AND gate. Another cause is tank accessories leakage, which involves damage to valves and poor seals around the manhole. Continue to develop the FTA until all of its branches have been ended by undeveloped or BEs. In the proposed fault tree, there are a totally of 70 events. Thus, 22 IEs and 48 BEs are the baseline element that contributes to the occurrence of fire accident and threatens storage tank protection. To better arrange the fault tree (Fig 3), we depicted transfer gates represented by triangles for three IEs: leakage in the storage tank, lighting sparks and static sparks.

### Application of FTA-SPA

At first, considering the fire accident as an undesirable event, the fault tree was fully drawn. Subsequently, both qualitative and quantitative evaluations can be conducted. For quantitative analysis, expert elicitation and SPA have been carried out to calculate the failure probability of the BEs. Experts from many professions must decide the failure probability of events based on their knowledge and experience. Integrated expert judgments were used in risk assessment under specified conditions to make the final result more accurate.

The results of the five chosen expert’s measurements are illustrated in Table 3. The ordered results in Table 3 can to assist experts in qualifying the measures of BEs. Experts were consulted about the probability of occurrence and non-occurrence. As shown in Table 4, each BE’s connection number is determined by weighted expert rating techniques.

**Table 3:** Selected expert profiles and their weighting score.

Experts	Job title	Years of related experience	Educational degree	Age	Weighting of expert (*W*_*i*_)
Expert 1	(4)	(4)	(3)	(3)	14/59 = 0.2373
Expert 2	(2)	(2)	(4)	(1)	9/59 = 0.1525
Expert 3	(3)	(4)	(4)	(2)	13/59 = 0.2204
Expert 4	(3)	(5)	(3)	(4)	15/59 = 0.2542
Expert 5	(2)	(2)	(3)	(1)	8/59 = 0.1356
					Total = 1

**Table 4:** Connection number of the BEs.

Basic event	Connection number	Basic event	Connection number
(*BE*_*i*_)	$\left[\begin{array}{ccc} A & B & C \end{array}\right]$	(*BE*_*i*_)	$\left[\begin{array}{ccc} A & B & C \end{array}\right]$
*BE*_01_	$\left[\begin{array}{ccc} 0.7220 & 0.1000 & 0.1780 \end{array}\right]$	*BE*_25_	$\left[\begin{array}{ccc} 0.7280 & 0.0754 & 0.1966 \end{array}\right]$
*BE*_02_	$\left[\begin{array}{ccc} 0.6678 & 0.2102 & 0.1220 \end{array}\right]$	*BE*_26_	$\left[\begin{array}{ccc} 0.5763 & 0.2305 & 0.1932 \end{array}\right]$
*BE*_03_	$\left[\begin{array}{ccc} 0.4848 & 0.1746 & 0.3406 \end{array}\right]$	*BE*_27_	$\left[\begin{array}{ccc} 0.5709 & 0.1146 & 0.3145 \end{array}\right]$
*BE*_04_	$\left[\begin{array}{ccc} 0.5492 & 0.2237 & 0.2271 \end{array}\right]$	*BE*_28_	$\left[\begin{array}{ccc} 0.6022 & 0.1604 & 0.2374 \end{array}\right]$
*BE*_05_	$\left[\begin{array}{ccc} 0.3729 & 0.3560 & 0.2712 \end{array}\right]$	*BE*_29_	$\left[\begin{array}{ccc} 0.6022 & 0.0979 & 0.2999 \end{array}\right]$
*BE*_06_	$\left[\begin{array}{ccc} 0.2966 & 0.4915 & 0.2119 \end{array}\right]$	*BE*_30_	$\left[\begin{array}{ccc} 0.6126 & 0.0896 & 0.2978 \end{array}\right]$
*BE*_07_	$\left[\begin{array}{ccc} 0.8356 & 0.0576 & 0.1068 \end{array}\right]$	*BE*_31_	$\left[\begin{array}{ccc} 0.8110 & 0.1017 & 0.0873 \end{array}\right]$
*BE*_08_	$\left[\begin{array}{ccc} 0.9000 & 0.0383 & 0.0617 \end{array}\right]$	*BE*_32_	$\left[\begin{array}{ccc} 0.8021 & 0.1062 & 0.0917 \end{array}\right]$
*BE*_09_	$\left[\begin{array}{ccc} 0.8780 & 0.0548 & 0.0672 \end{array}\right]$	*BE*_33_	$\left[\begin{array}{ccc} 0.7021 & 0.0604 & 0.2375 \end{array}\right]$
*BE*_10_	$\left[\begin{array}{ccc} 0.9203 & 0.0239 & 0.0558 \end{array}\right]$	*BE*_34_	$\left[\begin{array}{ccc} 0.5564 & 0.1229 & 0.3207 \end{array}\right]$
*BE*_11_	$\left[\begin{array}{ccc} 0.5204 & 0.1475 & 0.3321 \end{array}\right]$	*BE*_35_	$\left[\begin{array}{ccc} 0.6355 & 0.1437 & 0.2208 \end{array}\right]$
*BE*_12_	$\left[\begin{array}{ccc} 0.6898 & 0.0873 & 0.2229 \end{array}\right]$	*BE*_36_	$\left[\begin{array}{ccc} 0.3068 & 0.5305 & 0.1627 \end{array}\right]$
*BE*_13_	$\left[\begin{array}{ccc} 0.5034 & 0.2017 & 0.2949 \end{array}\right]$	*BE*_37_	$\left[\begin{array}{ccc} 0.6417 & 0.0917 & 0.2666 \end{array}\right]$
*BE*_14_	$\left[\begin{array}{ccc} 0.3678 & 0.1644 & 0.4678 \end{array}\right]$	*BE*_38_	$\left[\begin{array}{ccc} 0.6397 & 0.0833 & 0.2770 \end{array}\right]$
*BE*_15_	$\left[\begin{array}{ccc} 0.6949 & 0.0966 & 0.2085 \end{array}\right]$	*BE*_39_	$\left[\begin{array}{ccc} 0.5938 & 0.0688 & 0.3374 \end{array}\right]$
*BE*_16_	$\left[\begin{array}{ccc} 0.6667 & 0.0604 & 0.2729 \end{array}\right]$	*BE*_40_	$\left[\begin{array}{ccc} 0.6729 & 0.1492 & 0.1780 \end{array}\right]$
*BE*_17_	$\left[\begin{array}{ccc} 0.6584 & 0.1374 & 0.2042 \end{array}\right]$	*BE*_41_	$\left[\begin{array}{ccc} 0.7119 & 0.1805 & 0.1076 \end{array}\right]$
*BE*_18_	$\left[\begin{array}{ccc} 0.5188 & 0.1521 & 0.3291 \end{array}\right]$	*BE*_42_	$\left[\begin{array}{ccc} 0.8034 & 0.1110 & 0.0856 \end{array}\right]$
*BE*_19_	$\left[\begin{array}{ccc} 0.4813 & 0.2063 & 0.3124 \end{array}\right]$	*BE*_43_	$\left[\begin{array}{ccc} 0.7220 & 0.1144 & 0.1636 \end{array}\right]$
*BE*_20_	$\left[\begin{array}{ccc} 0.6896 & 0.0979 & 0.2125 \end{array}\right]$	*BE*_44_	$\left[\begin{array}{ccc} 0.5373 & 0.2565 & 0.2062 \end{array}\right]$
*BE*_21_	$\left[\begin{array}{ccc} 0.6562 & 0.1084 & 0.2354 \end{array}\right]$	*BE*_45_	$\left[\begin{array}{ccc} 0.6938 & 0.0688 & 0.2374 \end{array}\right]$
*BE*_22_	$\left[\begin{array}{ccc} 0.6390 & 0.1034 & 0.2576 \end{array}\right]$	*BE*_46_	$\left[\begin{array}{ccc} 0.5563 & 0.1229 & 0.3208 \end{array}\right]$
*BE*_23_	$\left[\begin{array}{ccc} 0.6168 & 0.1916 & 0.1916 \end{array}\right]$	*BE*_47_	$\left[\begin{array}{ccc} 0.7334 & 0.0979 & 0.1687 \end{array}\right]$
*BE*_24_	$\left[\begin{array}{ccc} 0.6647 & 0.0916 & 0.2437 \end{array}\right]$	*BE*_48_	$\left[\begin{array}{ccc} 0.7475 & 0.1644 & 0.0881 \end{array}\right]$

According to the concept of proposed approaches, the result of the storage tank evaluation is received as follows:

$$\begin{array}{l} \mu (BE_{01})=(0.2373,0.1525,0.2204,0.2542,0.1356)*\left[\begin{array}{ccc} 0.8 & 0.1 & 0.1\\ 0.6 & 0.1 & 0.3\\ 0.7 & 0.1 & 0.2\\ 0.7 & 0.1 & 0.2\\ 0.8 & 0.1 & 0.1 \end{array}\right]*\left[\begin{array}{l} 1\\ i\\ j \end{array}\right]\\ \mu (BE_{02})=(0.2373,0.1525,0.2204,0.2542,0.1356)*\left[\begin{array}{ccc} 0.7 & 0.2 & 0.1\\ 0.8 & 0.1 & 0.1\\ 0.6 & 0.2 & 0.2\\ 0.6 & 0.3 & 0.1\\ 0.7 & 0.2 & 0.1 \end{array}\right]*\left[\begin{array}{l} 1\\ i\\ j \end{array}\right] \end{array}$$ (14)

**Fig 4:**
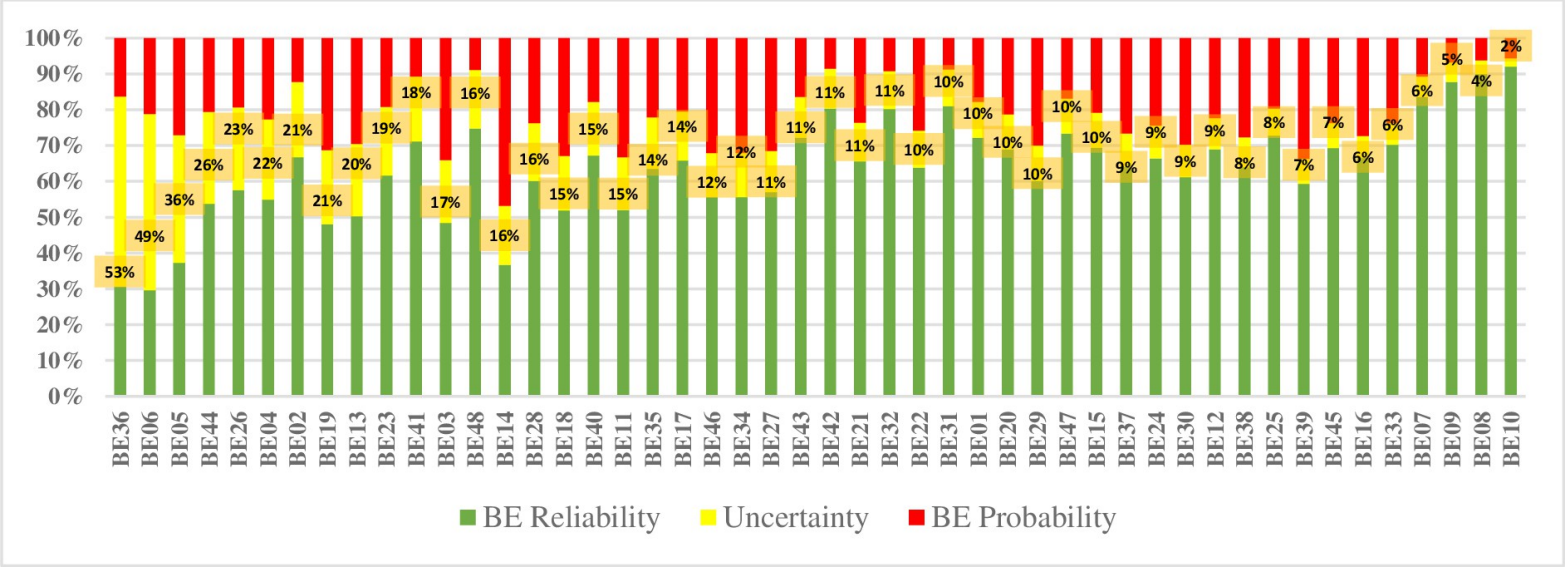
The connection number related to uncertainty of BEs.

These connection numbers are used as BE probabilities to quantify the probability of TE. The results obtained by the SPA noted that the failure probability of the BE14, BE03, BE39, BE11, BE18, BE46, BE34, BE27, BE19 and BE29 are the top 10 most crucial underlying causes in fire accident fault tree. Therefore, it is necessary to pay enough attention to reduce the occurrence. As can be seen from Table 4, BE10, BE08, BE09, BE07, BE31, BE42, BE32 and BE48 pose high reliability. Fig 4 indicates the BEs from highest to lowest connection number related to uncertainty, respectively.

Using the estimated data, and the BEs’ connection degree and their connection number can work out the probability of TE is as follow:

$$\mu_{TE}=\left(\sum_{i=01}^{03}\mu ({BE}_{i})+(\sum_{i=04}^{05}\mu ({BE}_{i})*\mu ({BE}_{06}))+\sum_{i=07}^{14}\mu ({BE}_{i})+\sum_{i=15}^{16}\mu ({BE}_{i})+(\sum_{i=17}^{19}\mu ({BE}_{i})*\sum_{i=20}^{22}\mu ({BE}_{i}))+\sum_{i=23}^{26}\mu ({BE}_{i})\right)*\left(\sum_{i=27}^{37}\mu ({BE}_{i})+(\sum_{i=38}^{42}\mu ({BE}_{i})*\sum_{i=43}^{45}\mu ({BE}_{i}))+\sum_{i=46}^{48}\mu ({BE}_{i})\right)$$ (15)

According to Eq (15) and the operation of algorithm rules, the connection number of the TE is received as follow:

$$\mu_{TE}=0.388+0.354i+0.258j$$ (16)

The risk analysis for storage tank operation is separated into three parts, denoted by the symbols A, B and C. To determine the storage tank operation level by selecting a value *i*, and the degree of correlation ranges from [-1 to 1]. Thus, a particular valve approach that correlates to the following degree of correlation with the appropriate assessment level is introduced:

$$\mu =\left\{ \begin{array}{c} {}[-1\;-0.333]\;fire\;accident\\ {}[-0.333\;0.333]\;general\;safety\\ {}[0.333\;1]\;safe\;condition \end{array}\right.$$ (17)

In the present study, the risk of storage tank operation under different conditions is evaluated by the equipartition principle. When *i* = 0 and *j* = −1, so *μ* = 0.130. The result shows that the access level of this storage tank operation is general safety. According to Eq (16), the probability of a tank fire is 2.58E-1/year, which is lower than the reliability of the safety state of 3.88E-1/year. Given that the difference in degree *i* influences the system’s operation, and in the worst scenario, *i* = −1, a tank fire accident may have gotten a level of 6.12E-1/year. However, the reliability is just 3.88E-1/year. These results that represent the probability value of fire accidents in the current storage tank could explain the overall chances of their occurrence. Adopting a positive mindset, *i* = 1, eliminates the uncertainty of the storage tank fire and results in the regular operation of the storage tank components, increasing the reliability to 7.42E-1/year. Regarding these ranges, we can evaluate an accident scenario. As previously stated, *i* is the coefficient of uncertainty. The uncertainty value can be derived from the certainty value for each condition. The value of *i* has two kinds, reflecting that it is a change process of the reliability of the storage tank operation from a worse scenario to the best scenario.

### Minimum cut sets and ranking

There are 48 BEs linked by AND/OR gate in the FTA of fire accident (Fig 3). The proposed fault tree provides 812 MCSs for 48 BEs including 238 MCSs of second-order, 409 MCSs of third-order and 165 MCSs of fourth-order. These results indicate that the BE combination how leads to fire accidents. For example, in the second-order, 238 times various pair BEs can lead to the top event. Finally, the sum of all MCSs is equal to 812 orders.

Generally speaking, the frequency of occurrence is higher when the MCS order is lower [70]. Therefore, by considering the highest failure probability, the top 50 MCSs of second-orders that combinate faults leading to fire accidents are listed in Table 5. From this table, we find that BE14 (Existence of heat sources near the farm tank) is the ignition source in the thirteen most essential MCSs. Also, the second most crucial ignition sources are BE16 (Wearing non-antistatic clothing), BE11 (Vehicles without flame arresters) and BE03 (Using non-explosion tools). The leakage source comes mainly from BE46 (Drain valve failure), BE34 (Corrosion serious of the tank wall), BE27 (Tank aging), BE29 (Poor welding quality) and BE30 (A natural disaster like an earthquake) in 36 of the 50 most important MCSs.

**Table 5:** Top MCSs in fire accident fault tree.

MCS	Involved BEs	MCS	Involved BEs
*C*_01_	*BE*_14_ *BE*_33_	*C*_26_	*BE*_11_ *BE*_37_
*C*_02_	*BE*_14_ *BE*_31_	*C*_27_	*BE*_11_ *BE*_46_
*C*_03_	*BE*_14_ *BE*_32_	*C*_28_	*BE*_11_ *BE*_34_
*C*_04_	*BE*_14_ *BE*_47_	*C*_29_	*BE*_03_ *BE*_29_
*C*_05_	*BE*_14_ *BE*_37_	*C*_30_	*BE*_16_ *BE*_33_
*C*_06_	*BE*_14_ *BE*_30_	*C*_31_	*BE*_24_ *BE*_46_
*C*_07_	*BE*_14_ *BE*_29_	*C*_32_	*BE*_24_ *BE*_34_
*C*_08_	*BE*_14_ *BE*_27_	*C*_33_	*BE*_24_ *BE*_27_
*C*_09_	*BE*_14_ *BE*_48_	*C*_34_	*BE*_03_ *BE*_37_
*C*_10_	*BE*_14_ *BE*_35_	*C*_35_	*BE*_24_ *BE*_30_
*C*_11_	*BE*_14_ *BE*_34_	*C*_36_	*BE*_03_ *BE*_27_
*C*_12_	*BE*_14_ *BE*_46_	*C*_37_	*BE*_24_ *BE*_29_
*C*_13_	*BE*_14_ *BE*_28_	*C*_38_	*BE*_12_ *BE*_46_
*C*_14_	*BE*_16_ *BE*_30_	*C*_39_	*BE*_12_ *BE*_34_
*C*_15_	*BE*_16_ *BE*_46_	*C*_40_	*BE*_03_ *BE*_46_
*C*_16_	*BE*_16_ *BE*_34_	*C*_41_	*BE*_03_ *BE*_34_
*C*_17_	*BE*_16_ *BE*_27_	*C*_42_	*BE*_12_ *BE*_27_
*C*_18_	*BE*_16_ *BE*_29_	*C*_43_	*BE*_25_ *BE*_46_
*C*_19_	*BE*_11_ *BE*_30_	*C*_44_	*BE*_25_ *BE*_34_
*C*_20_	*BE*_11_ *BE*_33_	*C*_45_	*BE*_12_ *BE*_30_
*C*_21_	*BE*_11_ *BE*_29_	*C*_46_	*BE*_25_ *BE*_27_
*C*_22_	*BE*_03_ *BE*_33_	*C*_47_	*BE*_12_ *BE*_29_
*C*_23_	*BE*_11_ *BE*_27_	*C*_48_	*BE*_15_ *BE*_46_
*C*_24_	*BE*_03_ *BE*_30_	*C*_49_	*BE*_15_ *BE*_34_
*C*_25_	*BE*_16_ *BE*_37_	*C*_50_	*BE*_15_ *BE*_27_

The importance of analysis is used to find the MCS that played a significant role in the occurrence of the fire accident. The F-VI for the top 50 MCSs of the fire accident fault tree was calculated using Eq (13). Based on F-VI calculation; these MCSs are ranked as shown in Table 6. From Table 6, the result shows that the top 5 most important MCS which require pay attention are *BE*_14_*BE*_33_, *BE*_14_*BE*_31_, *BE*_14_*BE*_32_, *BE*_14_*BE*_47_ and *BE*_14_*BE*_37_.

**Table 6:** FVI-based importance ranking of the top 50 MCSs in fire accident fault tree.

MCS	*PC*_*i*_(*x*)	$I_{i}^{F-V}$	Rank	MCS	*PC*_*i*_(*x*)	$I_{i}^{F-V}$	Rank
*C*_01_	4.158E-01	1.61E+00	1	*C*_26_	3.518E-01	1.36E+00	15
*C*_02_	4.115E-01	1.60E+00	2	*C*_27_	3.517E-01	1.36E+00	15
*C*_03_	4.089E-01	1.59E+00	3	*C*_28_	3.517E-01	1.36E+00	15
*C*_04_	4.051E-01	1.57E+00	4	*C*_29_	3.505E-01	1.36E+00	15
*C*_05_	3.982E-01	1.54E+00	5	*C*_30_	3.499E-01	1.36E+00	15
*C*_06_	3.961E-01	1.54E+00	5	*C*_31_	3.488E-01	1.35E+00	16
*C*_07_	3.920E-01	1.52E+00	6	*C*_32_	3.487E-01	1.35E+00	16
*C*_08_	3.827E-01	1.48E+00	7	*C*_33_	3.481E-01	1.35E+00	16
*C*_09_	3.820E-01	1.48E+00	7	*C*_34_	3.478E-01	1.35E+00	16
*C*_10_	3.785E-01	1.47E+00	8	*C*_35_	3.472E-01	1.35E+00	16
*C*_11_	3.782E-01	1.47E+00	8	*C*_36_	3.469E-01	1.34E+00	17
*C*_12_	3.782E-01	1.47E+00	8	*C*_37_	3.461E-01	1.34E+00	17
*C*_13_	3.690E-01	1.43E+00	9	*C*_38_	3.452E-01	1.34E+00	17
*C*_14_	3.657E-01	1.42E+00	10	*C*_39_	3.452E-01	1.34E+00	17
*C*_15_	3.656E-01	1.42E+00	10	*C*_40_	3.450E-01	1.34E+00	17
*C*_16_	3.656E-01	1.42E+00	10	*C*_41_	3.450E-01	1.34E+00	17
*C*_17_	3.654E-01	1.42E+00	10	*C*_42_	3.448E-01	1.33E+00	18
*C*_18_	3.642E-01	1.41E+00	11	*C*_43_	3.429E-01	1.33E+00	18
*C*_19_	3.584E-01	1.39E+00	12	*C*_44_	3.428E-01	1.33E+00	18
*C*_20_	3.568E-01	1.38E+00	13	*C*_45_	3.419E-01	1.33E+00	18
*C*_21_	3.560E-01	1.38E+00	13	*C*_46_	3.411E-01	1.32E+00	19
*C*_22_	3.543E-01	1.37E+00	14	*C*_47_	3.411E-01	1.32E+00	19
*C*_23_	3.532E-01	1.37E+00	14	*C*_48_	3.389E-01	1.31E+00	20
*C*_24_	3.530E-01	1.37E+00	14	*C*_49_	3.388E-01	1.31E+00	20
*C*_25_	3.528E-01	1.37E+00	14	*C*_50_	3.375E-01	1.31E+00	20

## Conclusions

In this study, a complete fault tree and its analysis related to the fire accident in a methanol storage tank was executed to illustrate the advantages of the proposed approach. Toward calculating conventional FTA, the lack of failure data was a deficiency. Therefore, the obtained results of the SPA added a new value to BEs and tackled the problem. The results showed that such an operation technique could be more effortless than other evaluation methods and more operable when failure data are absent.

One of the most significant findings of the present study was that FTA and SPA could be complementary methodologies. SPA describes the functionality that the system should follow. In contrast, FTA describes the fault scenarios where the system should work safely and predictably. The FTA without BEs’ failure probability is only good at presenting how the system is resistant. However, FTA-SPA uses this good feature and covers FTA limitations by applying mathematical calculations. This work was shown that the combination of FTA and SPA could be applied successfully for the fire risk assessment of storage tanks.

The proposed approach established in the present study can be adjusted to various systems with limited manipulation in future works. However, some questions have arisen due to this study and require further investigation. First, it is best in future work to compare the proposed method with other integrated methods, e.g., Fuzzy FTA. Emphasis has been put on assessing the possible consequences (e.g., explosion) by using bow-tie analysis with the proposed approach that will be studied in future papers.
